# The effectiveness of patient-centered care vs. usual care in type 2 diabetes self-management: A systematic review and meta-analysis

**DOI:** 10.3389/fpubh.2022.994766

**Published:** 2022-10-28

**Authors:** Kainat Asmat, Khairunnisa Dhamani, Raisa Gul, Erika Sivarajan Froelicher

**Affiliations:** ^1^Shifa College of Nursing, Shifa Tameer-e-Millat University, Islamabad, Pakistan; ^2^Department of Physiological Nursing, School of Nursing and Department of Epidemiology & Biostatistics, School of Medicine, University of California, San Francisco, San Francisco, CA, United States

**Keywords:** education and counseling, HbA1c, meta-analysis, type 2 diabetes, self-management, patient-centered care

## Abstract

**Background:**

Patient-centered care in diabetes self-management might be a significant factor in improving health outcomes of adults with type 2 diabetes, yet the supporting evidence is inadequate. This review aimed at assessing the effectiveness of patient-centered self-management care interventions on glycemic control (HbA1c) and self-care behaviors compared with usual care.

**Methods:**

CINAHL, PubMed, Cochrane Library, Google Scholar, and the HEC Digital Library were searched for studies in English language that assessed patient-centered self-management educational and/or behavioral interventions in adults aged 18 years or older with type 2 diabetes from 2005 to 2020. Interventional studies with at least 3 months of follow-up and reporting on self-care outcomes such as glycemic control (HbA1c) and self-care behaviors including diet control, physical activity, foot care, and medication adherence were included.

**Results:**

Of 168 identified records, 24 were found eligible comprising 20 RCTs and four QESs with total 4,083 participants. The meta-analysis involved 19 RCTs that provided enough information for a pooled estimate of HbA1c. Compared with the control group, patient-centered self-management interventions significantly lowered HbA1c, −0.56 (95% CI −0.79, −0.32). Stratified analysis for HbA1c with respect to various aspects of intervention showed larger effects in interventions employing both educational and behavioral components, −0.66 (95% CI −0.97, −0.34); spanned over shorter (<03 months) duration, −0.85 (95% CI −1.28, −0.43); administered by nurses, −0.80 (95% CI −1.44, −0.16); and delivered in community settings −0.70 (95% CI −1.14, −0.26).

**Conclusion:**

This systematic review provided evidence supporting the effectiveness of patient-centered self-management care interventions in improving glycemic control and self-care behaviors in adults with type 2 diabetes and identified key features of intervention contributing toward success.

## Introduction

Diabetes mellitus (DM) is one of the major health problems of the 21st century due to its growing prevalence and the risk of increased morbidity and mortality (1, 2). In 2021, the International Diabetes Federation (IDF) estimated that one in 10 adults aged 20–79 years have DM, equivalent to 537 million people worldwide (3). The IDF report showed a higher prevalence in the Middle East and North Africa, especially in low- and middle-income countries where three in four adults are affected. (4) More than 90% of patients with DM have type 2, simply known as type 2 diabetes. Micro- and macrovascular complications resulting from hyperglycemia in type 2 diabetes affect individuals' functional capacity, quality of life, and demand for healthcare services, with a significant economic impact on healthcare system and national economies (5, 6). The rise in type 2 diabetes is driven by socioeconomic, demographic, environmental, and genetic factors (7, 8). Age, sedentary lifestyle, metabolic syndrome, systematic low-grade inflammation, and insulin resistance are all well-known risk factors for type 2 diabetes. Type 2 diabetes is also linked with obesity, and there is a strong immune-metabolic connection between the two diseases (9). Furthermore, in low- to middle-income countries, a poor healthcare system, inaccessibility to healthcare centers, inequality in the provision of healthcare services, gender disparity, and poor socioeconomic conditions, as well as low level of education, are all contributing factors (10).

The growing prevalence of type 2 diabetes and the associated health and economic burden must be addressed on an urgent basis. The only pillar is delaying disease progression and avoiding diabetes-related complications, which may lead to better health and economic outcomes for patients, families, society, and the healthcare system as a whole. Evidence based guidelines suggest that progression of type 2 diabetes can be delayed and serious complications might be avoided by adopting a healthy lifestyle through improvement in self-care behaviors with medication as required (11). Therefore, type 2 diabetes is also known as a self-managed condition because the majority of the care is provided by patients themselves (12).

However, self-management requires patients' full commitment and capability to perform self-care activities, including healthy dietary habits, physical activity, blood glucose monitoring, and regular intake of medicines. Patients need to make a concerted and self-motivated effort toward adoption of a healthy life style as pharmacotherapy alone cannot achieve these goals (13). Also, type 2 diabetes is associated with complexity because there are multiple risk factors mostly involving a behavioral or social component that the individuals, their family, or society must struggle hard to implement. Therefore, a patient-centered behavioral or social approach would make a long-lasting impact toward effective disease management. Patient-centered care (PCC) has been recognized as a desirable attribute of healthcare since the late 1980s when the concept ‘patient-centeredness' was introduced. Patient-centeredness is characterized by utilization of a bio-psycho-social perspective, which means focusing on patients and honoring their preferences as a holistic being, rather than adopting a biomedical perspective that focuses on the disease (14). The shift from a biomedical model to a patient-centered care model may necessitate more effective patient engagement, collaboration on an individual care plan, and motivation of patients to adopt self-management behaviors (15). Therefore, PCC is characterized as a care that is tailored to the patients' specific needs, values, and preferences (16). PCC is an important factor in the self-management of type 2 diabetes and is associated with improved health outcomes such as quality of life and self-care behaviors in this population (17). The American Diabetes Association (ADA), in their consensus report, also advocated PCC to enhance patient engagement in self-care activities for type 2 diabetes self-management (18). Moreover, PCC improved patient activation in terms of knowledge, motivation, confidence, and skills, as well as better illness perception and a lower level of distress, in people with type 2 diabetes (19).

PCC in type 2 diabetes is defined as a purposefully designed holistic care intervention that provides information and skills needed for effective self-management of the disease based on patients' preferences to achieve optimum glycemic control by improving self-care behaviors in addition to medication (20). Self-management education is the major component of PCC that, according to the WHO, provides the basis for management of the disease (21). The literature supports that up to 8% of DM-associated complications can be reduced through proper self-management education (22). Behavioral intervention is another major component of PCC in type 2 diabetes self-management. The American Association of Diabetes Educators (AADE) suggests to prepare patients for behavior modification by equipping them with the necessary skills to improve their self-care behaviors. According to the AADE, seven parameters of self-care behaviors are healthy diet, regular physical activity, regular blood sugar monitoring, medication adherence, effective problem-solving approach, resilient coping skills, and risk reduction behaviors (23). Studies have shown behavioral interventions aimed at self-care activities significantly improved glycemic control, reduced diabetes-associated complications, and contributed to enhanced quality of life of patients with diabetes (24). A meta-analysis published in 2003 has demonstrated the effectiveness of behavioral interventions in improving self-care outcomes and the overall health status in patients with type 2 diabetes (25).

Given the increasing prevalence of type 2 diabetes worldwide and the risk of increased morbidity and mortality, PCC can play a crucial role in effective self-management of the disease. Therefore, an updated systematic review of PCC would give a better understanding of whether this care approach is associated with better clinical outcomes. This review aimed at assessing the effectiveness of PCC employing educational and behavioral interventions on glycemic control and self-care behaviors in adults with type 2 diabetes compared with usual care.

## Methods

### Search strategy

**Figure 1:**
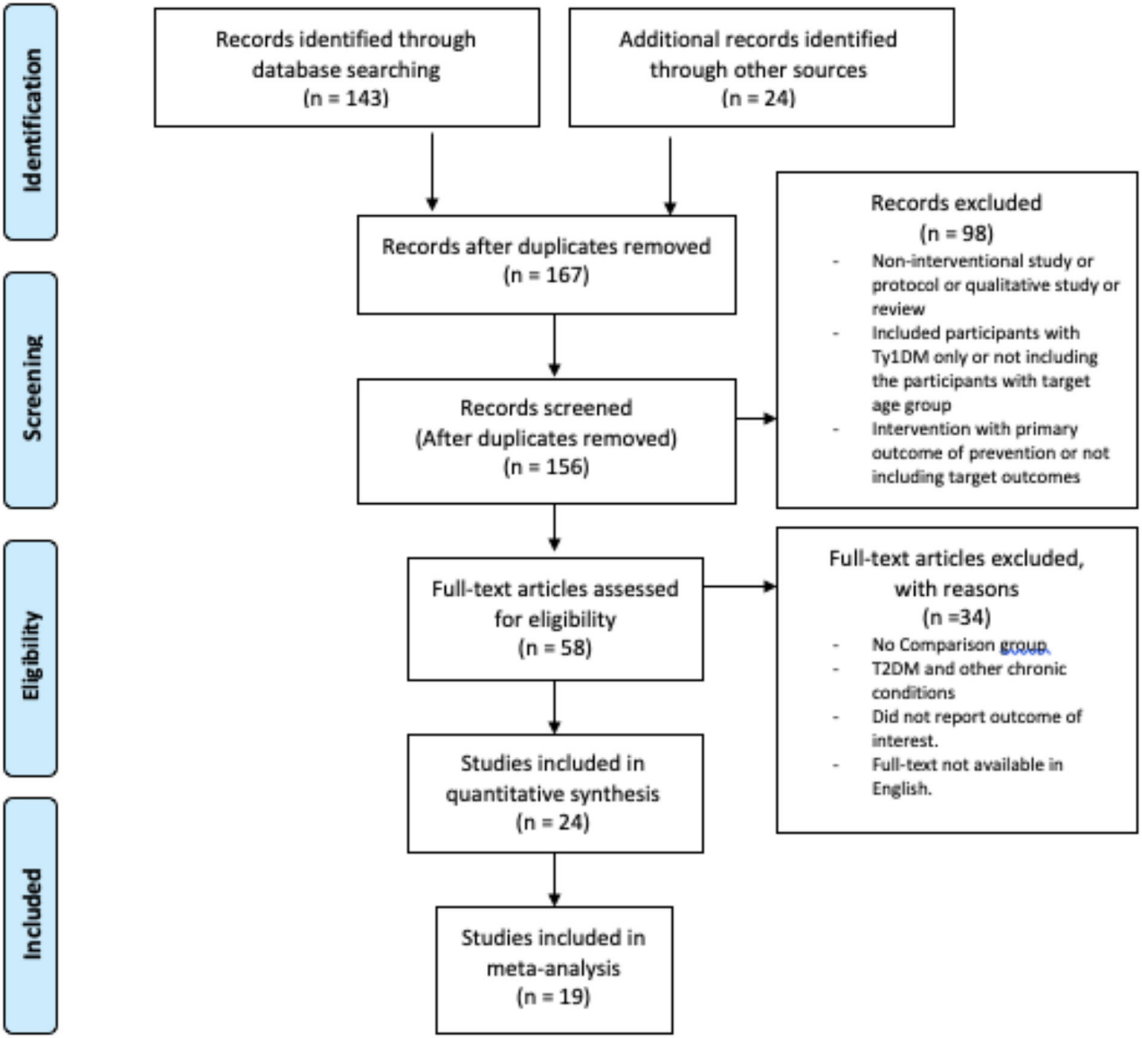
PRISMA flow chart for study selection and reasons for exclusion.

The literature search was performed in CINAHL, PubMed, Cochrane Library, Google Scholar, and the HEC Digital Library for studies in English language published between 1 January 1990 and 30 October 2020. 1 January 1990 has been selected as the search initiation date because the term patient-centered care/patient centeredness have been introduced in the literature in the late 1980s (26). The Medical Subject Headings (MeSH) terms and TIAB terms used were “Diabetes Mellitis, Type 2” OR “Type 2 Diabetes,” OR “Type II Diabetes,” AND “Patient-Centered-Care,” OR “Person-Oriented-Care,” OR “Holistic Care,” AND “Self-Management,” OR “Self-Care” AND “Glycated Hemoglobin A” OR Glycemic Control” or “HbA1c” AND “Self-Care Behaviours” OR Self-Care Activities”. The retrieved titles and abstracts were evaluated for relevance. Articles found relevant were reviewed as full text for consideration of inclusion in this review by completing the eligibility form based on inclusion criteria. In addition to systematic database searches, a manual search was performed to find studies in reference lists of relevant articles and reviews. Duplicates were removed with the help of Mendeley Reference Manager. With the exception of one study conducted in 1998 (27), the initial search in the databases retrieved records between 2005 and 2020. Because a study carried out in the 1990s would offer a limited scope due to changes in lifestyle, the search duration was reduced to 2005–2020. This review was planned, conducted, and reported in accordance with Preferred Reporting Items for Systematic Reviews and Meta-Analysis (PRISMA) guidelines (28). The PRISMA flowchart for selection of the studies and reasons for exclusion are presented in Figure 1.

### Inclusion and exclusion criteria

Studies were found eligible if they meet the following inclusion criteria: (1) type of studies as interventional studies including randomized controlled trials (RCTs) and quasi-experimental studies (QESs); (2) type of participants as adults (≥ 18 years) diagnosed with type 2 diabetes for at least last 6 months; (3) type of intervention as patient-centered care intervention for diabetes self-management with educational and/or behavioral component provided in any setting, by any method, by any provider, for any contact time, and with at least 3 months of follow-up; (4) comparison intervention as usual care or standard care; and last, (5) type of outcomes involving glycemic control (HbA1c) as the primary outcome, and self-care behaviors including diet control, physical activity, foot care, and medication adherence as secondary outcomes.

The following studies were excluded: (1) review, (2) non-intervention study, (3) qualitative study, (4) protocol, (5) patients with type 1 diabetes only, (6) adult patients with type 2 diabetes and with other chronic conditions, and (7) patients younger than 18 years targeted exclusively at prevention of type 2 diabetes.

### Data extraction

Data from the eligible studies were extracted and entered into an Excel sheet. Entered data were verified two times for accuracy and completeness. Discrepancies in the extracted data were discussed, and disagreements were adjudicated by reaching consensus.

### Quality assessment of individual studies

**Figure 2:**
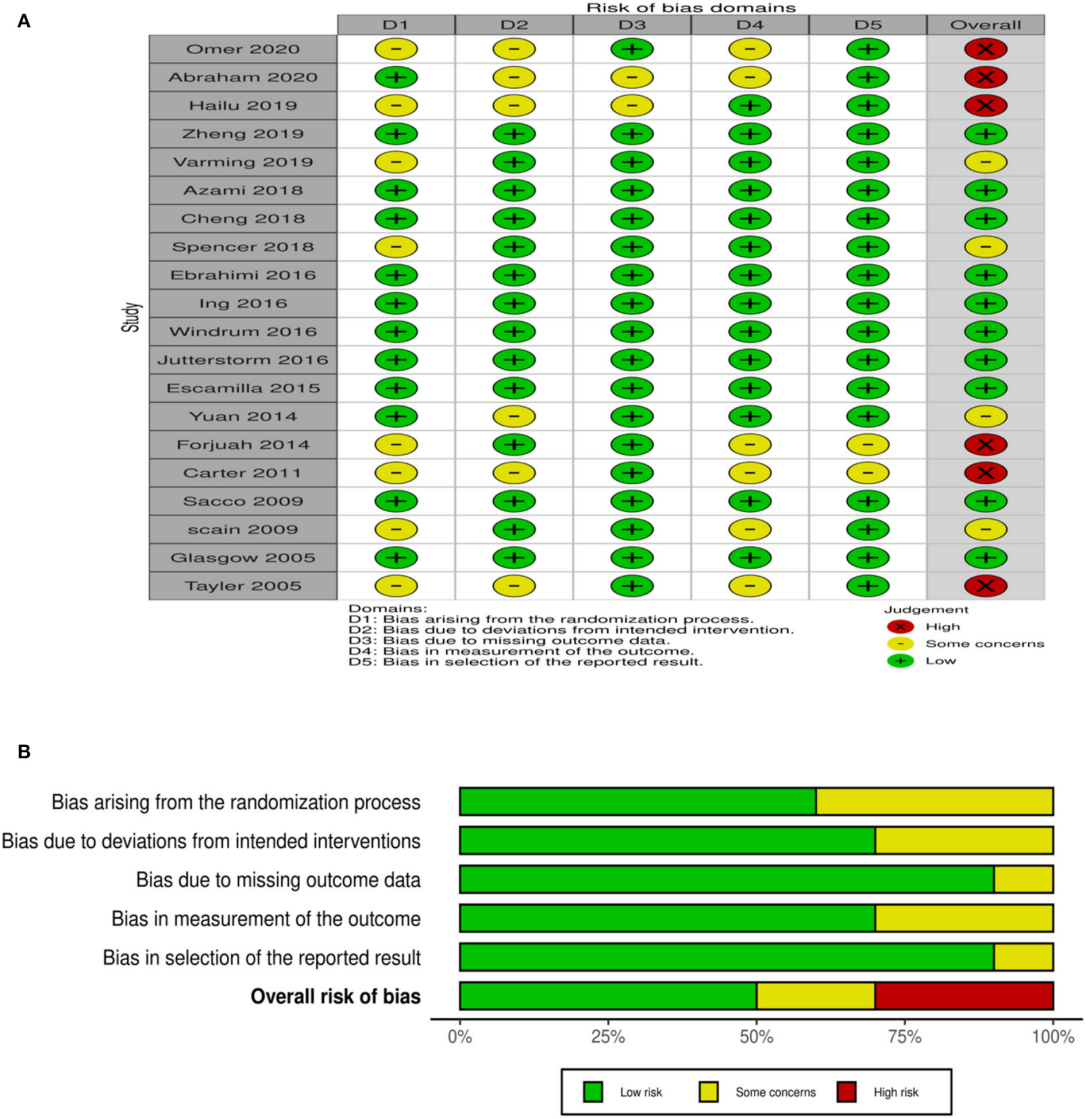
**(A)** Quality of RCTs assessed by Rob 2. **(B)** Risk of bias summary RCTs.

Individual studies were assessed for methodological quality by using the Cochrane Collaboration risk-of-bias assessment tool that yields a judgment for low, high, or unclear risk (expressing some concerns). Authors of the studies were contacted to request additional information. Rob 2 (version 2 of the Cochrane risk-of-bias tool for randomized trials) was used for RCTs (29). Quality of included RCTs was assessed on five domains of risk of bias in the randomization process, deviation in intended intervention, missing outcome data, measurement of outcome, and selection of the reported result, as shown in Figure 2 with a summary plot.

**Figure 3:**
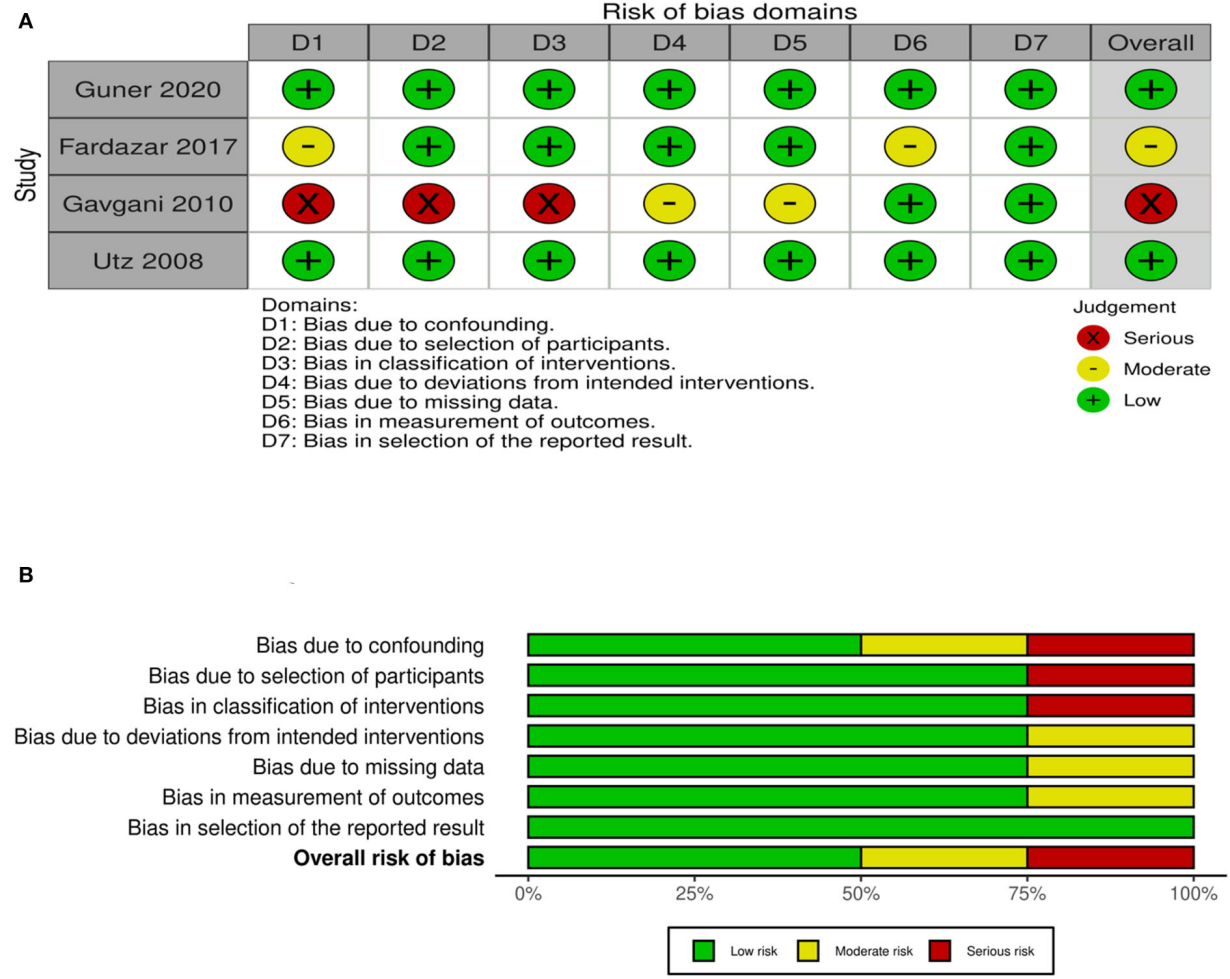
**(A)** Quality of QESs assessed by using ROBINS-1. **(B)** Risk of bias summary QESs.

For quality assessment of QESs, Risk Of Bias In Non-randomized Studies of Interventions (ROBINS-I) was used (30). Quality of QESs assessed by using ROBINS-1 on seven domains is given in Figure 3 with a summary plot.

### Statistical analysis

A meta-analysis was performed using REVMAN 5.4.1 to calculate the magnitude of pooled effect size for change in HbA1c, the primary outcome (31). Of 24 included studies, 22 reported on HbA1c, which included 19 RCTs and three QESs. However, only RCTs were included in the meta-analysis due to their optimal validity and causal inference. Data entered in REVMAN involved final values of mean and standard deviation of HbA1c for the experimental group and control group, and the number of participants in each group. Standardized mean difference of HbA1c between experimental and control groups and 95% confidence intervals were calculated for estimation of effect size. I^2^ statistics were used to estimate statistical heterogeneity among studies. The random effect model was applied on more than 50% heterogeneity (32). To further explore sources of heterogeneity, stratified analysis was performed based on key aspects of intervention, including (a) component of intervention (educational vs. educational and behavioral), (b) duration of intervention (<3 months vs. 3–6 months vs. > 6 months), (c) provider of intervention (nurse vs. other professional vs. ≥ 2 disciplines), and (d) setting of intervention (hospital vs. community vs. combined hospital and community).

## Results

### Characteristics of studies

For this review, 24 studies met the eligibility criteria. The included studies were published between 2005 and 2020 where the majority was published between 2015 and 2020. A majority of the studies were RCTs, accounting for 83%. In total, 4,083 participants were involved, with the mean age of 56.1 years (range 18–69 years). The study population involved patients with type 2 diabetes, with a mean duration of disease of 7.5 years (range 06–12.9 years). The sample size of a single study ranged from 22 to 886 involving both male and female patients. The characteristics of included studies are presented in Table 1.

**Table 1:** Characteristics of 24 studies included in the review.

**No**.	**Author and year**	**Country**	**Study design**	**No. of participants recruited / at follow up**	**Intervention**						**Length of follow p**	**Clinical indicators/ outcomes measured**	**Results**
					**Theoretical basis**	**Mode of delivery**	**Provider**	**Setting**	**Components**	**Duration**			
1	Glasgow et al. (33)	USA	RCT*	886/733	NR***	Face to Face	Physician	Hospital	Educational behavioral	06 months	12 months	HbA1c Foot Care	Significant improvement in HbA1c and Foot Care in experimental group.
2	Tayler et al. (34)	Canada	RCT	40/39	Supportive care model	Face to face	Nurse physician	Community	Educational behavioral	03 months	04 months	HbA1c	Small but non-significant improvement in HbA1c in experimental group.
3	Scain et al. (35)	Brazil	RCT	104/104	NR	Face to face	Nurse	Hospital	Educational	04 weeks	12 MONTHS	HbA1c Weight	Significant improvement in HbA1c in experimental group. Weight improved significantly, and similarly, in both groups.
4	Sacco et al. (36)	USA	RCT	62/48	NR	Telephone	Psychologist	Community	Educational	06 months	06 months	HbA1c Diet Exercise Foot care	Non-significant improvement in HbA1c. Significant improvement in diet, exercise and foot care in experimental group.
5	Carter et al. (37)	USA	RCT	74/47	NR	Online	Nurse	Community	Educational Behavioral	NR	09 Months	HbA1c BMI	Significant improvement in: HbA1c, BMI in experimental group.
6	Forjuoh et al. (38)	USA	RCT	376/263	NR	Face to face and online	Physician	Hospital and community	Educational Behavioral	06 weeks	12 months	HbA1c BMI Foot care	Non-significant improvement in HbA1c, BMI, foot care in experimental group.
7	Yuan et al. (39)	China	RCT	88/76	NR	Face to face	Nutritionist	Community	Educational	08 weeks	03 months	HbA1c Weight	Significant improvement in HbA1c and body weight in the experimental group
8	Escamilla et al. (40)	USA	RCT	211/148	NR	Face to face	Community health workers	Community	Educational Behavioral	12 months	18 Months	HbA1c Weight	Significant improvement in HbA1c in experimental group. Non-significant improvement in weight.
9	Ebrahimi et al. (41)	Iran	RCT	106/103	Empowerment Model	Face to face	Nurse endocrinologist nutritionist	Hospital	Educational Behavioral	08 weeks	03 months	HbA1c	Significant improvement in HbA1c in experimental group.
10	Jutterström et al. (42)	Sweden	RCT	195/171	Hernandez theory of integration	Face to face	Nurse	Hospital	Educational Behavioral	06 months	12 months	HbA1c BMI	Significant improvement in HbA1c and BMI in experimental group.
11	Windrum. et al. (43)	UK	RCT	203/203	NR	Face to face	Physician	Hospital	Educational	03 weeks	12 months	HbA1c	Significant improvement in HbA1c in experimental group
12	Azami et al. (44)	Iran	RCT	142/136	Social Cognitive Theory	Face to face	Nurse	Hospital	Educational Behavioral	12 weeks	06 months	HbA1c Weight	Significant improvement in HbA1c and body weight in experimental group.
13	Abraham et al. (45)	India	RCT	80/41	NR	Face to face and telephone	Physician	Hospital	Educational Behavioral	08 weeks	03 months	HbA1c	Significant improvement in HbA1c in experimental group.
14	Cheng et al. (46)	China	RCT	242/201	Empowerment Model	Face to face	Nurse	Hospital	Educational Behavioral	06 weeks	05 months	HbA1c Diet Control	Non-significant improvement in HbA1c in experimental group. Significant improvement in diet control.
15	Zheng et al. (47)	China	RCT	60/60	NR	Face to face	Physician	Hospital	Educational Behavioral	02 weeks	03 months	HbA1c	Significant improvement in HbA1c in experimental group.
16	Ing et al. (48)	USA	RCT	65/38	NR	Face to face	Physician Certified diabetes educator Community worker Pharmacist	Hospital	Educational Behavioral	03 months	06 months	HbA1c Diet control Physical activity Foot care	Significant improvement in HbA1c, diet control, physical activity, and foot care in experimental group
17	Varming et al. (49)	Denmark	RCT	154/97	NR	Face to face	Nurse	Hospital	Educational Behavioral	03 months	06 months	HbA1c Diet control Physical activity Foot care Medication adherence	Non-significant improvement in HbA1c, diet control, physical activity, foot care and medication adherence.
18	Spencer et al. (50)	Canada	RCT	222/147	Empowerment Model	Face to face	CHW	Community	Educational Behavioral	06 months	18 months	HbA1c	Significant improvement in HbA1c in experimental group
19	Omer et al. (51)	UAE	RCT	218/164	NR	Online *via* WhatsApp	Pharmacist	Community	Educational	06 Months	06 Months	HbA1c	Significant improvement in HbA1c in experimental group
20	Hailu et al. (52)	Ethiopia	RCT	220/142	NR	Face to face	Nurse	Hospital	Educational Behavioral	06 months	09 months	Diet Control Physical activity Foot care	Significant improvement in diet control, physical activity, and foot care in intervention group
21	Utz et al. (53)	USA	QES**	22/21	Social Cognitive Theory	Face to Face	Certified Diabetes Educator (CDE)	Community	Educational Behavioral	08 weeks	06 months	HbA1c Diet Exercise Foot care Medication Adherence	Significant improvement in HbA1c in experimental group. Non-significant improvement in Diet, exercise, foot care and medication adherence.
22	Gavgani et al. (54)	Iran	QES	32/30	Information Motivation and Behavioral skill Model	Face to face	Physician	Hospital	Educational Behavioral	02 weeks	02 months	HbA1c Diet Exercise Foot care	Significant improvement in HbA1c, Diet, exercise in experimental group. Foot care was more than the control group but not statistically significant.
23	Fardazar et al. (55)	Iran	QES	180/180	attribution theory	Face to face	Physician	Hospital	Educational	NR	03 months	Diet, Physical activity, Foot care	Significant improvement in diet, physical activity and foot care in experimental group.
24	Guner et al. (56)	Turkey	QES	101/101	NR	Face to face and Telephone	Nurse Physician	Community	Educational Behavioral	06 months	06 months	HbA1c BMI	Significant improvement in HbA1c and BMI in experimental group.

*Randomized controlled trial; **quasi-experimental study; ***not reported.

### Characteristics of intervention

Interventions of all studies were compared with usual care. Usual care majorly involved consultation with the physician; having blood sugar, blood pressure, and weight checked; and getting scheduled for the next appointment, which sometimes included verbal or written guidance on lifestyle changes. Overall, 37.5% of (nine of 24) studies reported on the theoretical model used for design and implementation of DM self-management intervention (34, 41, 42, 44, 46, 50, 53–55). In total, two studies (44, 53) based their intervention on the social cognitive theory; three studies (41, 46, 50) on the empowerment model; one study based on the supportive care model (34), one based on information motivation and behavioral skill model (54), one based on the Hernandez theory of integration (42), and one based on the attribution theory (55). The mean duration of intervention was 12 weeks, ranging from 2 weeks (54) to 12 months (40). A wide range of intensity of intervention was reported ranging from 15 to 20 min over a day to 1,530 min over 12 months. The majority of the (18 of 24) studies administered both educational and behavioral components of intervention. The follow-up duration ranged from 2 months (54) to 18 months (40). A few (four of 24) studies used the multidisciplinary care approach involving two or more than two different members of the healthcare team delivering the intervention. The majority of studies involved interventions delivered at hospitals and some in community settings, whereas only one study reported administration of intervention both in hospitals and in community settings (38).

### Study outcomes

#### Primary outcome (glycemic control HbA1c)

**Figure 4:**
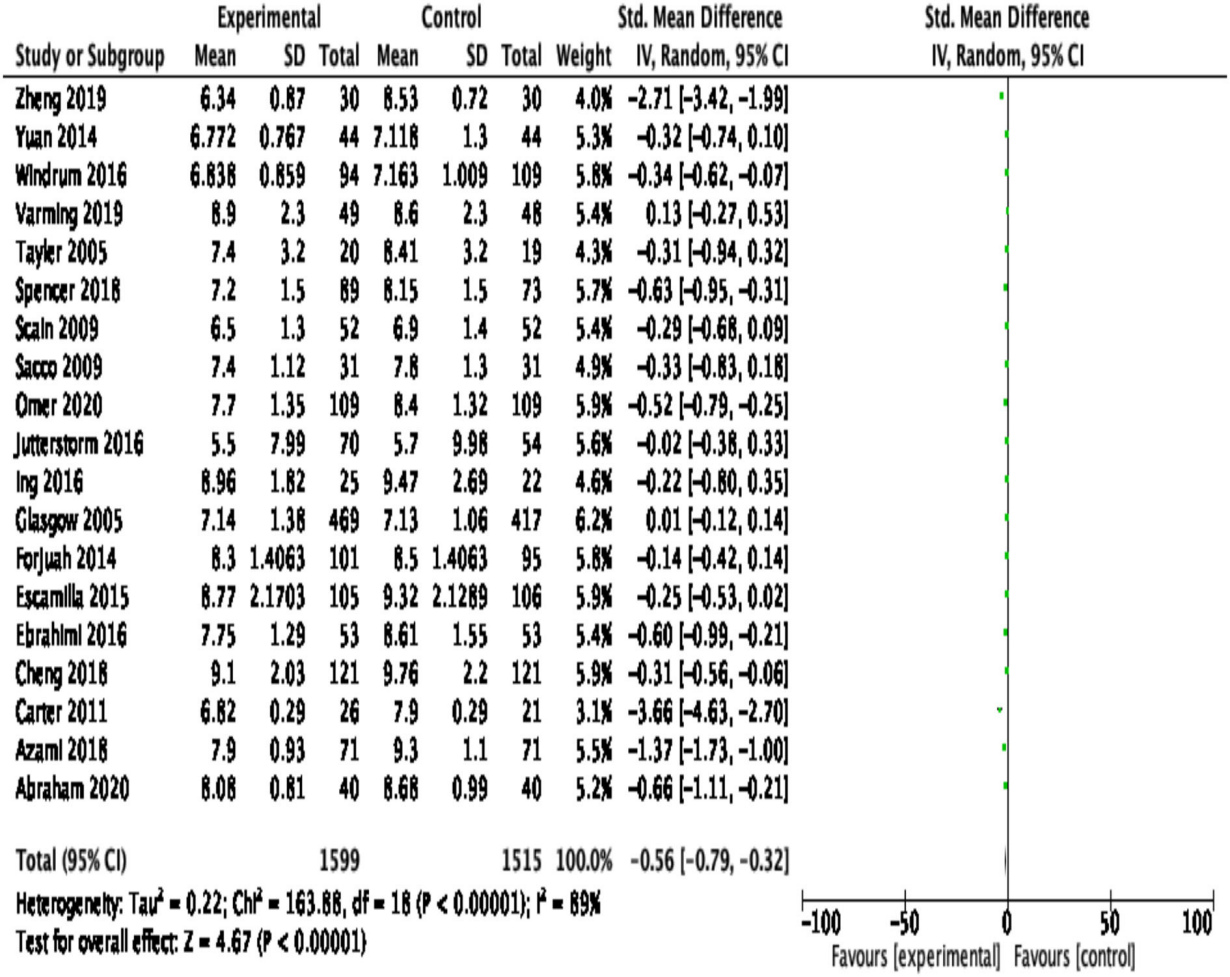
Forest plot of pooled effect size of HbA1c.

**Figure 5:**
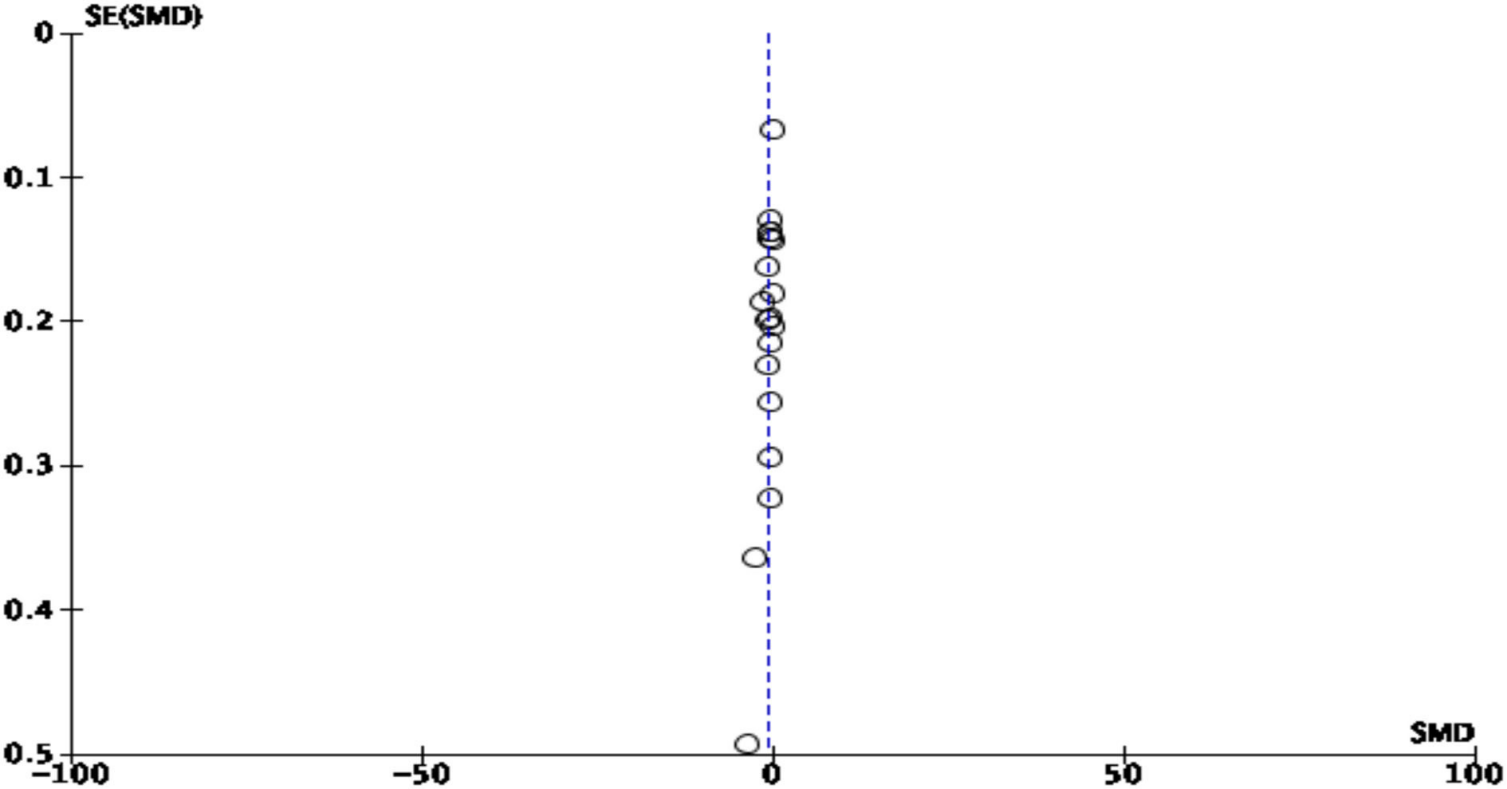
Funnel plot for publication bias in HbA1c effects.

HbA1c was reported as an outcome measure in 22 studies. The majority of the studies reported statistically significant reduction in HbA1c. Because of the significant heterogeneity (>50%) among studies, the random effect model was applied. At 95% CI with 3,114 participants in 19 RCTs, the magnitude of effect, −0.56 (95% CI −0.79, −0.32), was statistically significant (*p* < 0.00001), showing a substantial reduction in HbA1c in the experimental group compared with the control group. The pooled effect size of HbA1c is shown in Figure 4. The likelihood of publication bias among studies was measured using a Funnel plot, as illustrated in Figure 5. When there is no suspicion of publication bias, the observed studies are scattered symmetrically around the pooled effect size. Visual inspection of the funnel plot shows that the observed studies are clustered around the mean, displaying an equal distribution. However, there are gaps with no scattered points to the left and right of the mean, signifying some publication bias.

##### Stratified analysis based on components of intervention

**Figure 6:**
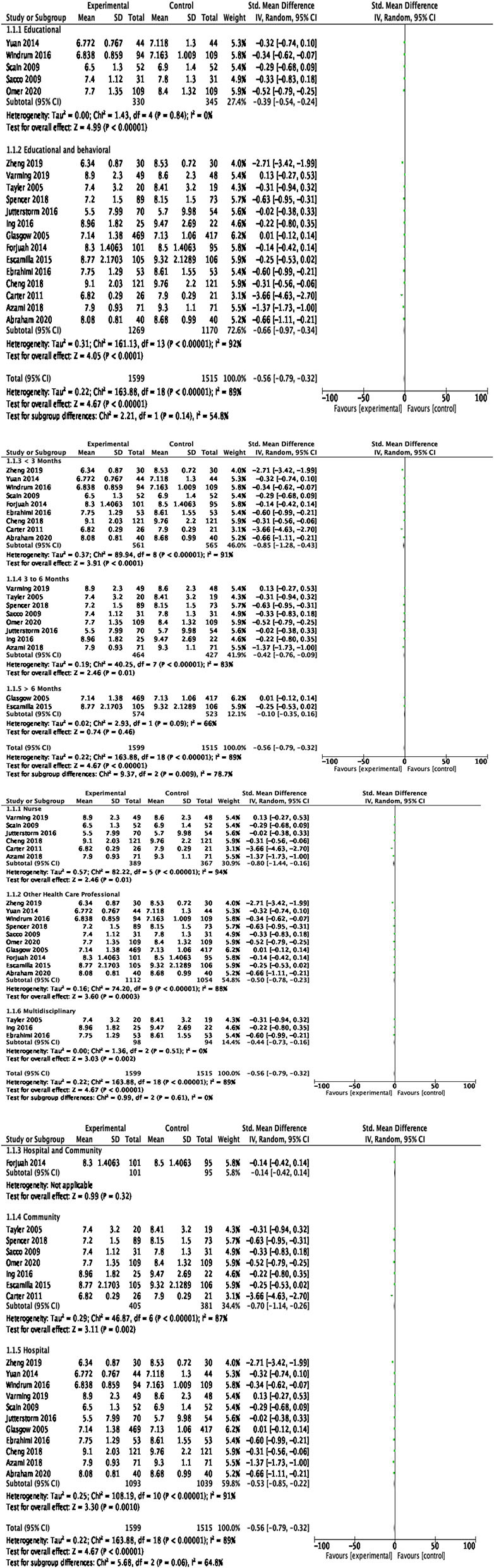
**(A)** Pooled effect size of HbA1c in studies sub-grouped by components of intervention; **(B)** pooled effect size of HbA1c in studies sub-grouped by duration of intervention; **(C)** pooled effect size of HbA1c in studies sub-grouped by provider of intervention; **(D)** pooled effect size of HbA1c in studies sub-grouped by setting of intervention.

Earlier, Gary TL et al. in their meta-analysis concluded that studies with the behavioral component of intervention were found more effective in reducing HbA1c than studies involving the educational component only (25). In this analysis, studies were sub-grouped into two components of intervention: (1) educational only and (2) combined educational and behavioral intervention. Pooled effect size indicated that studies with combined educational and behavioral components yielded larger effect size (−0.66; 95% CI −0.97, −0.34) than studies with the educational component only (−0.39; 95% CI −0.54, −0.24). Overall heterogeneity (I^2^) was 89%; therefore, the random effect model was applied (see Figure 6A).

##### Stratified analysis based on duration of intervention

Existing evidence suggested that a longer duration of intervention (> 6 months) showed significant reduction in HbA1c compared with a shorter duration (<6 months) (25) Considering the span of intervention, studies were sub-grouped into (1) studies with a duration of intervention <3 months, (2) 3–6 months, and (3) > 6 months. Pooled effect size indicated that studies with a shorter duration (<3 months) produced larger effect size (−0.85; 95% CI −1.28, −0.43) than studies with a duration of intervention of 3–6 months (−0.42; 95% CI −0.76, −0.09) and studies with a longer duration of intervention of > 6 months (−0.10; 95% CI −0.35, −0.16). Overall heterogeneity (I^2^) was 89%; therefore, the random effect model was applied (see Figure 6B).

##### Stratified analysis based on provider of intervention

Evidence suggests that a multidisciplinary team approach is more effective in improving HbA1c (57). Given this, a stratified analysis was performed by dividing studies into three groups based on the provider of intervention: (1) a nurse, (2) other healthcare professional such as a physician, nutritionist, pharmacist, or community health worker; and (3) multidisciplinary team members (≥ 2 disciplines). Pooled effect size indicated that studies involved a nurse as a provider of intervention produced larger effect size (−0.80; 95% CI −1.44, −0.16) than studies with other professional as s provider (−0.50; 95% CI −0.78, −0.23) and studies involving ≥ 2 disciplines (−0.44; 95% CI −0.73, −0.16). Overall heterogeneity (I^2^) was 89%; therefore, the random effect model was applied (see Figure 6C).

##### Stratified analysis based on setting of intervention

To see the effect of setting, a stratified analysis was performed by grouping studies into three categories: (1) hospital, (2) community, and (3) combined setting including both hospital and community. Studies with intervention delivered in community settings produced larger effect size (−0.70; 95% CI −1.14, −0.26) than those in hospitals (−0.53; 95% CI −0.85, −0.22) and combined settings (−0.14; 95% CI −0.42, −0.14). Overall heterogeneity was 89%; therefore, a random effect model was applied (see Figure 6D).

#### Secondary outcomes

Secondary outcomes included diet control, physical activity, foot care, and medication adherence. A total of 16 (35–40, 42, 44, 46, 48, 49, 52–56) studies reported on dietary outcomes including body mass index (BMI) and weight, where the majority (69%; 11 of 16) showed statistically significant improvement in diet control in the experimental group receiving PCC; seven studies (36, 48, 49, 52–55) reported on physical activity or exercise outcomes, where the majority (71%; five of seven) showed significant improvement in physical activity in the experimental group receiving PCC; nine studies (33, 36, 38, 48, 49, 52–55) reported on foot care outcome, where more than half (55%; five of nine) showed statistically significant improvement in foot care in the experimental group receiving PCC; and two studies (49, 53) reported on this outcome that showed non-significant improvement in medication adherence in the experimental group receiving PCC.

#### Summary of findings

Table 2 shows a summary of findings including the sum of available data on primary outcomes (HbA1c), magnitude of the intervention effect, and certainty of evidence utilizing the Group Reading Assessment and Diagnostic Evaluation (GRADE) approach (58).

**Table 2:** Summary of findings of HbA1c.

**Outcome**	**No. of Participants (No of studies & design)**	**Risk of bias**	**Inconsistency**	**Indirectedness**	**Imprecision**	**Publication bias**	**Overall Quality of Evidence**
HbA1C	3114 19 RCTs	Not serious	Serious	Not serious	Not serious	Not serious	Moderate ⊕⊕⊕○

Table 2 illustrates that in terms of risk of bias, none of the RCTs demonstrated a high risk in any of the five domains. Of the 19 RCTs, 10 had a low risk of bias; four had some concerns in one domain, indicating an unclear risk of bias; and five RCTs had high risk of bias, showing some concerns in multiple domains. There is inconsistency found in the results of HbA1c indicated by substantial heterogeneity. Variability in the social environment and conditions of delivery of intervention, which may not be discernible from published results, may be the reasons for this unexplained heterogeneity. With regard to indirectness, the patients, interventions, and comparison in the included studies provided direct evidence to the clinical question. However, the authors examined some variations in the mode and delivery of intervention. In terms of imprecision, the authors judged to have no serious imprecision. The included trials enrolled 4,113 patients, with some trials reporting significant results and others reporting non-significant results, most likely due to the small number of participants enrolled, which resulted in wide confidence intervals and no effects. Last, the authors did not find strong suspicion of publication bias since both the positive and negative results of trials were reported. Summarizing all, the authors judged the overall quality of the evidence as moderate. Future research focusing on PCC social and situational factors will likely have a significant impact on the confidence in effect estimate.

## Discussion

This review aimed at evaluating the effectiveness of patient-centered self-management care interventions on self-care outcomes of adults with type 2 diabetes compared with usual care. The most important indicator of optimum management of DM is glycemic control (HbA1c). Therefore, the primary outcome of this review was glycemic control (HbA1c), whereas changes in diet control, physical activity, foot care, and medication adherence were the secondary outcomes.

To estimate the overall effect of intervention, a meta-analysis was performed to calculate the magnitude of effect size for change in HbA1c. Pooled effect size indicated a statistically significant difference in HbA1c between experimental and control groups, −0.56 (95% CI −0.79, −0.32). The findings of this review are similar to the findings of previous meta-analysis by Gray et al. in 2003, which reported statistically significant reduction in HbA1c, −0.43 (95% CI −0.71, −0.15) (25). This review confirmed that the patient-centered self-management interventions are accompanied with a significant decrease in HbA1c. Since HbA1c is one of the important predictors of DM-associated complications and a key therapeutic goal toward its effective self-management, findings of this review have some important implications for contemporary practice. Evidence suggests that 21% of risk is reduced for any DM-associated complication and its related deaths with a 1% decline in HbA1c (59). Thus, reduction in HbA1c has clinical significance. However, unexplained heterogeneity due to variations in the social environment and conditions of intervention administration may hamper meta-analysis results. Moreover, patient-centered self-management care interventions are influenced by multiple situational factors, such as marital status, socioeconomic conditions, and cultural norms. There is a need to explore such situational and sociocultural factors affecting patient-centered care provision.

To explore heterogeneity further, a stratified analysis for change in HbA1c was performed with regard to various key aspects of intervention to ascertain key elements that might contribute toward effective self-management of type 2 DM. In this review, interventions involving educational and behavioral components, spanned over a shorter (<3 months) duration, provided by nurses, and delivered in community settings were found more effective as indicated by larger effect sizes. Some findings of this review are contrary to those of the previous meta-analysis by Gary et al., where interventions that involved a longer duration (>3 months) and provided by physicians were found more effective. It appears that interventions with a longer duration may carry out an element of fatigue due to long contact times, which may produce lesser effect. A previous systematic review by Norris et al. (60) also found inconsistencies in the duration of intervention and its beneficial effects. There is a need to further explore the factors associated with the intervention duration demonstrating an area of research. Moreover, the finding of this review showing larger effects with nurses as providers of intervention emphasizes the importance that nurses are uniquely positioned to bring their expertise and knowledge toward effective self-management of type 2 diabetes. This again demonstrates an area of research to further investigate the clinical effectiveness of nurse-led interventions. The findings are consistent with those of a previous meta-analysis with regard to the setting of intervention confirming that interventions delivered in community settings are more effective. This confirms that community settings are the best place for patients living in neighborhood to form peer groups in order to gain diabetes knowledge, reinforce each other, and receive support for change in behavior.

With regard to secondary outcomes, this review indicated that patient-centered self-management care interventions are effective in improving patients' diet control, physical activity, and foot care. Diet control improved significantly in the majority of included studies, confirming the findings of a previous systematic review by Norris et al. (60) These findings are also consistent with Williams et al. stating that PCC improves dietary behaviors in patients with type 2 DM (17). The change in physical activity was also found effective, confirming the results of previous studies (61, 62). However, the effect on medication adherence was not found significant, which may be due to the reason that only two studies reported this outcome. These findings are not consistent with the study by Williams et al., which reported a significant association of PCC with medication adherence (17). This may necessitate an area of further exploration, which could help identify the key components of PCC interventions targeting medication adherence. Overall, this review provided with the evidence that PCC for self-management is effective in improving glycemic control and self-care behaviors in adults with type 2 diabetes.

## Limitations and strengths

The limitations of this review are as follows: including only studies in English language; selective reporting of the outcomes, which might have affected the findings; frequent methodological biases found in included studies; insufficient description of intervention in the included studies; and failure in reporting medications or any drug prescription information because medication intake may act as a confounder between the interventions and outcomes.

However, this review has several strengths: rigorous reviewing methods, a thorough search to capture all relevant information, explicit and reproducible eligibility criteria, and stratified analysis with answers to clinically relevant and important questions.

## Conclusion

Overall, this review provided with the evidence that PCC for self-management is effective in improving glycemic control and self-care behaviors in adults with type 2 diabetes. Some gaps were found that are needed to be addressed: (1) only few studies provided a thorough description with regard to the intervention including intensity, duration, length of follow-up, and theoretical background; (2) the behavioral component was not described in adequate detail with regard to the methods applied, and (3) medication adherence was reported only by a few studies.

## Data availability statement

The original contributions presented in the study are included in the article/supplementary material, further inquiries can be directed to the corresponding author.

## Author contributions

KA: study conception and design, literature retrieval, and drafting of the manuscript. KD: data analysis and interpretation and critical revision of the manuscript. RG: study conception, critical revision, and final approval of the manuscript. EF: conceptualizing the review process, data analysis and interpretation, critical revision, and final approval of the manuscript. All authors contributed to the article and approved the submitted version.

## Conflict of interest

The authors declare that the research was conducted in the absence of any commercial or financial relationships that could be construed as a potential conflict of interest.

## Publisher's note

All claims expressed in this article are solely those of the authors and do not necessarily represent those of their affiliated organizations, or those of the publisher, the editors and the reviewers. Any product that may be evaluated in this article, or claim that may be made by its manufacturer, is not guaranteed or endorsed by the publisher.

## Abbreviations

AADE: American Association of Diabetes Educators
ADA: American Diabetes Association
CINAHL: Cumulative Index to Nursing and Allied Health
DM: diabetes mellitus
GRADE: Grading of Recommendations, Assessment, Development and Evaluation
IDF: International Diabetes Federation
PCC: patient-centered care
QES: quasi-experimental study
RCT: randomized controlled trial
WHO: World Health Organization.
